# Progress in the Study of Vortex Pinning Centers in High-Temperature Superconducting Films

**DOI:** 10.3390/nano12224000

**Published:** 2022-11-13

**Authors:** Jian Zhang, Haiyan Wu, Guangzhen Zhao, Lu Han, Jun Zhang

**Affiliations:** 1 Key Laboratory for Water Quality and Conservation of the Pearl River Delta, Institute of Environmental Research at Greater Bay Area, Guangzhou University, Ministry of Education, Guangzhou 510006, China; 2 Key Laboratory of Spin Electron and Nanomaterials of Anhui Higher Education Institutes, Suzhou University, Suzhou 234000, China; 3 School of Pharmacy, Dali University, Dali 671000, China

**Keywords:** high-temperature superconducting films, vortex pinning, artificial pinning center, nanoparticle, nanocolumn

## Abstract

Since the discovery of high-temperature superconductors (HTSs), significant progress in the fabrication of HTS films has been achieved. In this review, we intend to provide an overview of recent progress in how and why superconductivity can be enhanced by introducing nanoscale vortex pinning centers. The comprehensive control of morphology, dimension, orientation and concentration of artificial pinning centers (APCs) and the principle of vortex pinning are the focus of this review. According to the existing literature, HTSs with the best superconductivity can be obtained when one-dimensional (1D) and three-dimensional (3D) nanoscale APCs are combined for vortex pinning.

## 1. Introduction

Superconductors are a class of materials with unique physical properties and high application value. Within critical parameters, superconductors have two major properties, namely: the zero-resistance effect and the Meissner effect. *T*_c_ (critical transition temperature), *B*_c_ (critical magnetic field), and *J*_c_ (critical current density) are the main critical parameters of superconductors. Currently, superconductors are classified into Type-I and Type-II superconductors. For the Type-I superconductor, there is only one critical magnetic field *B*_c_. However, for the Type-II superconductor, there are two critical magnetic fields, the lower *B*_c1_ and upper *B*_c2_. When *B*_c1_ > *B*, the superconductor remains in the Meissner state, completely expelling the magnetic flux from its interior. For *B*_c2_ > *B* > *B*_c1_, the magnetic flux starts penetrating the sample in the form of discrete bundles termed “flux lines” and the sample goes into the mixed state (or vortex state). When *B* > *B*_c2_, the superconductor comes into the normal state.

Usually, Type-II superconductors that exhibit superconducting states at ~30 K and above are called high-temperature superconductors (HTSs). They are first discovered in 1986 by J. G. Bednorz and K. A. Müller in the Ba-La-Cu-O system [1]. YBa_2_Cu_3_O_7-δ_ (YBCO) is the first HTS to be found with *T*_c_ above the liquid nitrogen temperature (LN_2_, 77 K) [2], and its discovery triggers a research boom in the field of superconductivity. Early research on HTS focused on exploring superconductors with higher *T*_c_, and scientists subsequently discovered several systems such as Bi_2_Sr_2_Ca_2_Cu_3_O_10+δ_ (BSCCO) [3], Tl_2_Ba_2_Ca_2_Cu_3_O_10+δ_ (TlBCCO) [4], HgBa_2_Ca_2_Cu_3_O_8+δ_ (HgBCCO) [5], all with *T*_c_~77 K and above. Since HTSs allow the use of LN_2_ as a cooling source, which is readily available and relatively inexpensive, they have a significant cost advantage over low-temperature superconductors (LTS) for future large-scale practical applications [6,7]. Mahesh Paidpilli et al. summarized in detail the various applications of HTSs in the high magnetic field in the United States at present in their review [8]. D. Uglietti summarized relevant research and developments in commercial HTS materials applied in large solenoids, accelerator dipoles, and high-field tokamaks [9]. In addition, other prominent applications of HTS including single-photon detectors based on superconducting nanowires (SNSPDs) [10], superconducting quantum interference devices (SQUIDs) [11,12,13] et al. have also been reported.

For HTS films, commonly used substrates include MgO, SrTiO_3_, LaAlO_3_, LaSrAlO_4_, YSZ (Yttria-Stabilized Zirconia), sapphire (with Ag, CeO_2_, and MgO buffer layers), and so on [14,15]. Due to the structural complexity, the phase composition of HTS films can vary depending on the deposition methods and parameters (e.g., substrate types, temperature, vacuum quality, accelerating voltage, etc.), resulting in different calcined phases. In addition, individual elements may be present in the form of metal oxides or compounds, and the generation of these additional phases increases the difficulty of preparing HTS films [16,17,18,19,20,21,22].

Controlling the morphology, dimension, orientation, and concentration of artificial pinning centers (APCs) has long been a desire of researchers and is crucial for the practical application of HTS. In this review, we have presented the development of materials engineering aspect that has been conducted over the last two decades to improve the current carrying capability of HTS films by introducing nanoscale APCs.

## 2. Natural Vortex Pinning Centers

**Figure 1:**
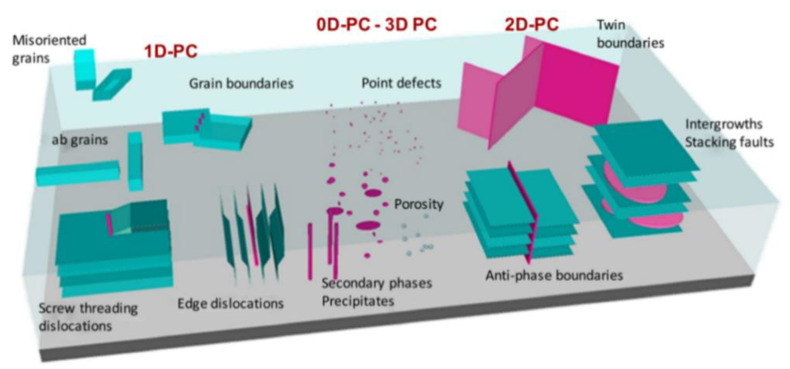
Schematic diagram of spontaneous vortex pinning centers of HTS films. Reprinted with permission from Ref. [33]. Copyright 2018, IOP Publishing Ltd.

HTSs spontaneously generate various types of crystal defects, impurities, and other non-ideal structures during the preparation process due to the complexity of structures. The generation of these defects can have an active role in vortex pinning and become spontaneous pinning centers. A schematic diagram of various spontaneous crystal defects in HTS films is shown in Figure 1. The defects which are naturally generated during the growth of HTS films can act as vortex pinning centers including point defects [23,24], voids, misfit dislocation, precipitates, grain boundaries [25,26], antiphase boundaries [27], twin boundaries [28,29], planar defects [30], and so on. Among them, grain boundary is one of the most common crystal defects in HTS films. At boundaries, where misaligned grains meet, atomic order is disrupted resulting in strain and dislocations, which provide pinning to the vortices.

However, the pinning efficiency of these naturally occurring defects is not sufficient to counter thermal fluctuations and maintain necessary *J*_c_ levels at high magnetic fields [31,32]. Therefore, the enhancement of *J*_c_ by introducing additional APCs into the superconducting film matrix has become a valuable research topic.

## 3. Artificial Pinning Centers (APCs)

To better pin the vortex at external magnetic fields, the HTS films must contain APCs with desired morphology, dimension, orientation, and concentration. Nanoscale APCs with lateral dimension approaching 2ξ (coherence length) on the order of a few nanometers in HTSs must be generated to suppress the dissipation of vortex motion. This has prompted extensive efforts in the past few decades or so and exciting results have been obtained in generating nanoscale APCs in HTS films. In this section, we introduced the research progress of different types and dimensions APCs in detail, and summarized the impact on superconducting performance.

### 3.1. Zero-Dimensional APCs (0D APCs)

The effect of ionic radii on the *T*_c_ of *RE*BCO has been documented in previous work [34]. It is well known that varied rare-earths have different ionic radii. The phenomenon that *T*_c_ varies linearly with ionic radius of *RE* ions has been detected and was attributed to strain-induced charge redistribution between the CuO_2_ planes and the charge reservoir (CuO-chains). Several rare-earth elements, including Sm, Eu, and Nd, have been doped in place of Y with various molar cationic ratios to enhance the vortex-pinning capabilities of YBCO films [35]. The Y atom in Y-Ba-Cu-O has been totally replaced in certain studies [35,36,37,38] by another rare-earth atom or a mixture of two or more rare-earth atoms, which has improved vortex pinning. Several combinations, including (Gd_0.8_Er_0.2_) [37] and (Nd_1/3_Gd_1/3_Eu_1/3_) [38], were published to determine whether the strain caused by lattice mismatch increased when mixtures of rare-earth elements were used instead of a single rare-earth element. Except for the situation when defects were random and unrelated, the enhancement was not notable in any circumstances. There have been attempts to substitute Tb, Ce, Pr, Nd, La, Co, Dy, and Eu at the Y site of YBCO and the *RE* site of *RE*BCO films [24,39,40,41,42]. The increased density of these substituent nanoprecipitates in doped *RE*BCO films compared to pristine *RE*BCO film led to elevated *J*_c_ and *F*_p_ values across a wide range of applied magnetic fields, which in turn led to stress field due to lattice mismatch between the phases in the resulting *RE*BCO films.

### 3.2. One-Dimensional APCs (1D APCs)

The idea of strain engineering has been applied to generate and control the morphology and dimension of APCs embedded in HTS films. According to the elastic strain energy model, the appropriate level of interfacial strain can act as a driving force for the self-assembly of 1D vortex pinning, controlling the morphology [43,44], dimensionality [45,46], orientation [47], and concentration [48]. Numerous studies have shown that 1D columnar APCs grown along the *c*-axis of *RE*Ba_2_Cu_3_O_7-δ_ films exhibit strong vortex pinning ability, resulting in high *J*_c_ when the applied magnetic field is along the *c*-axis direction [43,48,49,50].

**Figure 2:**
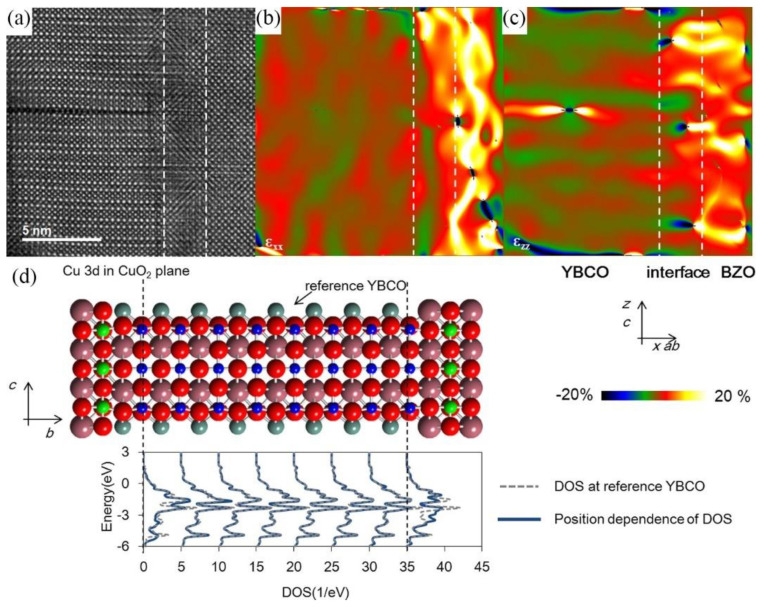
(**a**) HAADF image of the BaZrO_3_/YBCO heterointerface and (**b**) ε_xx_ and (**c**) ε_zz_ map of the heterointerface. (**d**) Dependence of pDOS of Cu 3d in the CuO_2_ plane on the heterointerface position. Reprinted with permission from Ref. [56]. Copyright 2017, American Chemical Society.

MacManus-Driscoll et al. [51] first reported the introduction of BaZrO_3_ secondary phase into YBCO films using the PLD (Pulsed Laser Deposition) technique to enhance the performance. It was found that BaZrO_3_ nanoparticles and nanocolumns produced significant *c*-axis orientation-related enhancement of *J*_c_ despite its random distribution in the YBCO matrix. Following the work of MacManus-Driscoll et al., 1D BaZrO_3_ APCs have been intensively investigated. In the subsequent report by Yamada et al. [52], the addition of YSZ (yttrium oxide stabilized zirconium oxide) to YBCO targets resulted in the formation of columnar BaZrO_3_ nanostructures in YBCO films and would leave a YBCO film matrix containing Ba defects. Self-assembly of vertical arrays of BaZrO_3_ phases is observed in this composite film. The vertical alignment of these self-assembled BaZrO_3_ columnar phases was hypothesized to be due to the preferential nucleation of impurity islands in the strain field above the impurity particles [53]. Physical property measurements showed that these self-assembled vertical BaZrO_3_ phase arrays resulted in strong pinning of vortices, especially when the applied magnetic field was along the *c*-axis direction. Goyal et al. [54] also reported enhanced pinning of BaZrO_3_/YBCO nanocomposite films along the *c*-axis direction. The BaZrO_3_/YBCO interface is strongly strained due to the high lattice mismatch of 7.7% between BaZrO_3_ and YBCO, which leads to the formation of a high defect density semi-coherent BaZrO_3_/YBCO heterointerface [55,56]. This defect is considered the source of the high pinning efficiency achieved at the 1D BaZrO_3_ magnetic flux pinning centers. Gutiérrez J et al. [56] performed HAADF-STEM observations and theoretical calculations on the interface region, where there are a large number of interfacial mismatch dislocations, as shown in Figure 2a–c. Another feature of the interface region is distortion, which denotes the local atomic displacement due to the interface atomic bonding. The matrix portion in the interface region is analyzed to discuss the superconductivity in the nanocomposites. Figure 2d shows a dependence of the pDOS (partial Density of States) of Cu 3d in the CuO_2_ plane on the interface position in the BaZrO_3_(2 layers)/YBCO(8 layers), which indicates that the pDOS of YBCO varied significantly in three unit-cell thick regions from the BaZrO_3_ columns.

**Figure 3:**
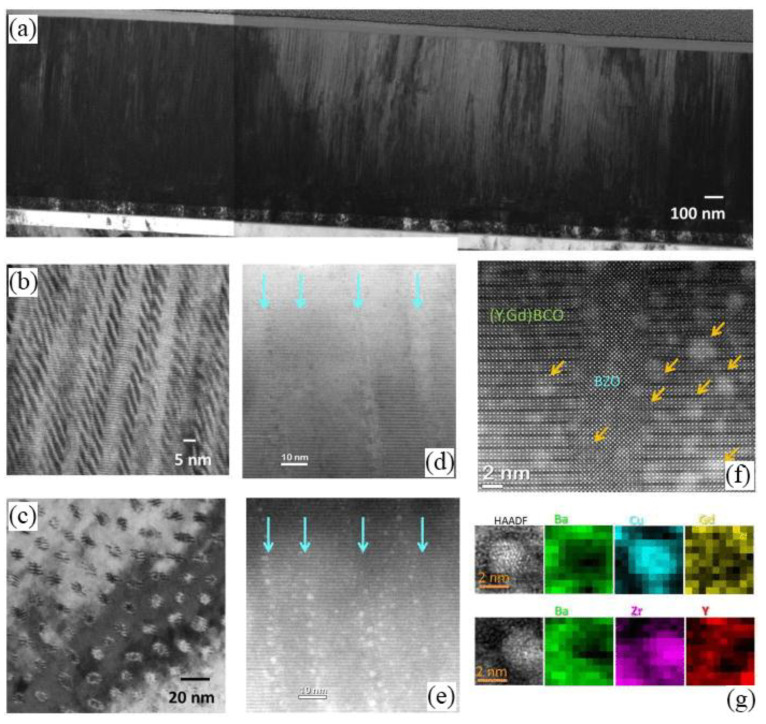
TEM image of Zr-added (Gd, Y)BCO superconductor tape. (**a**,**b**) cross-sectional TEM, (**c**) plan view TEM, (**d**) BF-STEM image, (**e**) HAADF image, (**f**) high-resolution image, and (**g**) element composition map of a nanoparticle. Reprinted with permission from Ref. [59]. Copyright 2015, AIP Publishing.

The force that resists the motion of vortices under the influence of Lorentz force is called the pinning force, whose density is termed as pinning forced density (*F*_p_). Researchers applied the MOCVD (metal-organic chemical vapor deposition) technique to demonstrate that the incorporation of BaZrO_3_ in relatively thick *RE*BCO films (1~2 μm) results in excellent *J*_c_ properties [57,58,59,60,61,62]. One of the nanocomposite films reached the highest recorded value of *F*_p, max_~1.7 TN/m^3^ (at 4.2 K) with *µ*_0_*H*_irr_ (irreversibility field)~14.8 T (at 77 K), which is much higher than the *µ*_0_*H*_irr_ of NbTi superconductor at 4.2 K (~11 T) [57]. In the cross-sectional and planar TEM images (Figure 3) of the heavily doped (Gd, Y)BCO films prepared by the MOCVD technique, self-assembled BaZrO_3_ nanocolumns with *c*-axis orientation can be observed. The critical current performance is excellent in the heavily doped films with *F*_p, max_ exceeding the value of 1 TN/m^3^ [59].

The search for new vortex pinning materials with smaller lattice mismatches with HTSs is the most effective and likely solution to improve superconductivity. In addition to BaZrO_3_, 1D-nanostructured materials such as BaSnO_3_ [63,64,65,66,67,68,69,70,71], BaTiO_3_ [72], BaHfO_3_ [63,73,74,75], YBa_2_(Nb/Ta)O_6_ [76,77,78,79] have also been successfully introduced into YBCO films using the PLD technique. These 1D APCs provide different degrees of vortex immobilization [63,68,73,74,75,77,78,80]. In all cases, the enhancement of *J*_c_ is more pronounced when the applied magnetic field is higher. Mele P et al. [71] reported a record *F*_p, max_ value of 28.3 GN/m^3^ for BaSnO_3_/YBCO nanocomposite films, reflecting the excellent *J*_c_ performance at that time. In addition, the double-perovskite material, YBa_2_NbO_6_ (YBNO), was also investigated as 1D APCs and introduced into the superconducting matrix [81]. In another study, Jha A. K. et al. [82] applied surface-modified target method to introduce YBa_2_NbO_6_ columns into YBCO films by controlling the rotational speed of the target to control the concentration of YBa_2_NbO_6_. YBa_2_NbO_6_ nanocolumns were observed to effectively enhance the *J*_c_ performance of YBCO films. Furthermore, *RE*_3_TaO_7_ and *RE*Ba_2_TaO_6_ were also proved to significantly enhance the *J*_c_ performance of *RE*BCO films [83,84], and the results indicated that lattice mismatch is a suitable condition to produce high pinning ability in the range of 5~12% [84].

Recently, BaHfO_3_(BHO) has sparked much interest among researchers as a very promising secondary phase APC, whose nano-inclusions in the form of columnar or spherical structures within the *RE*BCO matrix significantly improve the *J*_c_ values of *RE*BCO films deposited on single crystals and metal strips [85,86,87,88,89,90,91,92]. Tobita et al. [85] firstly reported that the BHO-doped GdBa_2_Cu_3_O_y_(GdBCO) film was deposited by PLD on the IBAD-MgO substrate. The most interesting feature of BHO nanocolumns addition was reported as *J*_c_ is undepressed by increasing thickness of the film. By using the LTG (Low-Temperature Growth) technique in PLD, BaHfO_3_/SmBCO films exhibit very high *F*_p, max_ (~28 GN/m^3^) at 77 K when *H* is parallel to the *c*-axis [89]. Even on metal tapes, the BaHfO_3_/GdBCO nanocomposite films exhibit a large *F*_p, max_ (~23.5 GN/m^3^) and a high irreversibility field (*µ*_0_*H*irr = 15.8 T) when *H* is parallel to the *c*-axis at 77 K [90]. In addition, BaHfO_3_ nanoparticles were also introduced into YBCO [91] and GdBCO [92] films using the CSD method, which improved the *J*_c_ of the nanocomposite films.

**Figure 4:**
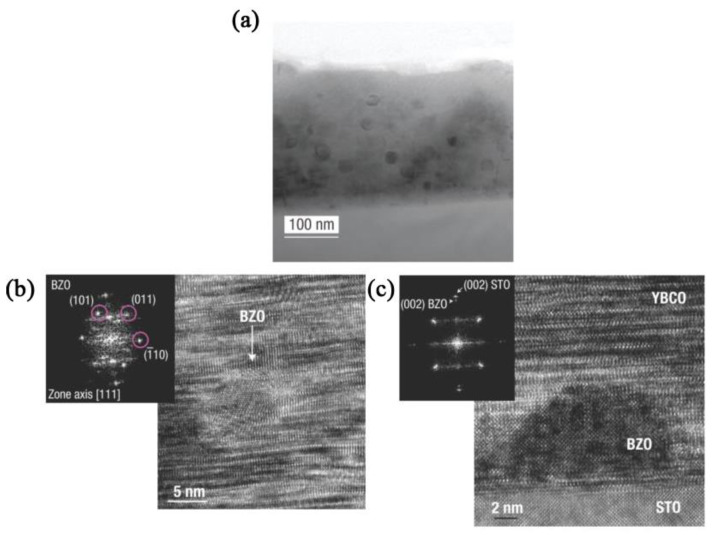
(**a**) TEM image of the BaZrO_3_/YBCO nanocomposite film. High-resolution TEM image of BaZrO_3_ nanoparticles nucleated in the YBCO matrix (**b**) and at the interface (**c**). Reprinted with permission from Ref. [93]. Copyright 2007, Nature Portfolio.

It is worth noting, 1D APCs are usually introduced to HTS film by PLD or MOCVD. However, the results in the preparation of BaZrO_3_/YBCO nanocomposite films by the CSD (chemical solution deposition) method are completely different [93]. In the preparation of HTS films by physical techniques represented by PLD, the nucleation of the superconducting matrix and the secondary phase is simultaneous, whereas in the preparation by CSD, the nucleation does not occur at the same time, leading to a different final phase morphology [94]. Gutiérrez, J. et al. [93] prepared BaZrO_3_/YBCO nanocomposite films using the CSD method and corresponding TEM images are shown in Figure 4. It can be noticed that non-columnar BaZrO_3_ spherical nanoparticles are produced in the matrix and surrounded by many crystal defects. This indicates that the difference in preparation methods can seriously affect the morphology of APCs. In contrast to the columnar BaZrO_3_ secondary phase, the formation of spherical BaZrO_3_ nanoparticles leads to thin films with isotropic pinning properties [95,96].

### 3.3. Two-Dimensional APCs (2D APCs)

The deposition of multilayer or quasi-multilayer film structures has also been used in HTS films to improve vortex pinning capabilities. For example, YBCO multilayer films have been prepared using the PLD technique (intermediate layers include: Ag [97], Pd [98], Y_2_O_3_ [99,100,101], BaZrO_3_ [102,103], SrRuO_3_ [104], SrTiO_3_ [105], LaCaMnO_3_ [106], YSZ [107], Y-211 [108], PrBa_2_Cu_3_O_x_ [109] and transition metals Ir [110], Ti, Zr, Hf [111]). The formation of Ba*M*O_3_ (*M* = Ti, Zr, Hf, Ir) phases can be observed after the addition of transition metal elements to YBCO films. Not only the *J*_c_ enhancement based on YBCO multilayers was observed from the physical property test results, but also the irreversibility lines moved to higher *H*-*T* regions [102].

**Figure 5:**
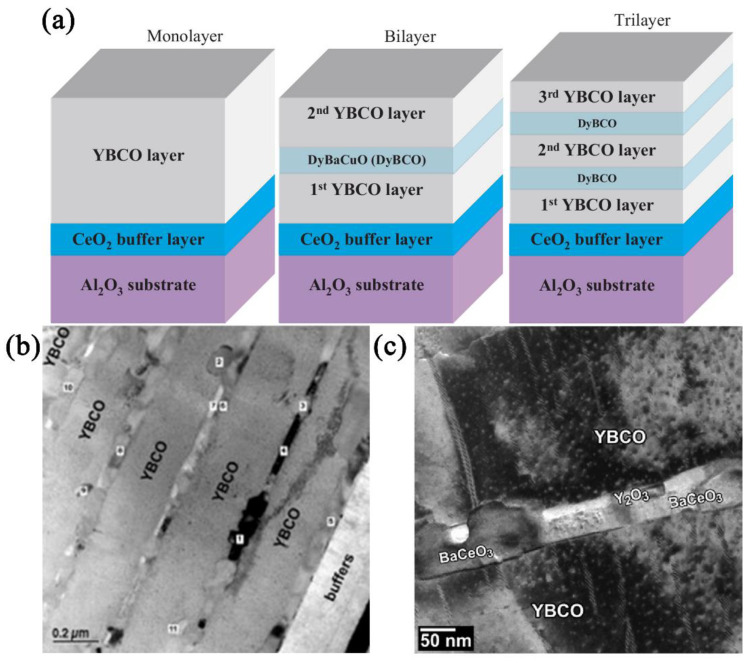
(**a**) Schematic illustration of the YBCO/DyBCO multilayers. Reprinted with permission from Ref. [113]. Copyright 2007, AIP Publishing. (**b**,**c**) Low and high magnification cross-section TEM micrograph of YBCO + BZO/CeO_2_ multilayer. Reprinted with permission from Ref. [114]. Copyright 2007, IOP Publishing.

K Develos-Bagarinao et al. [112,113] deposited relatively thick HTS films (~400–600 nm) with significantly improved surface morphology and *J*_c_ using a multilayered structure which alternated main layers of YBCO with intermediate DyBa_2_Cu_3_O_7−δ_ (DyBCO) layers on CeO_2_-buffered sapphire substrates by PLD. The DyBCO layer has a close lattice matching with YBCO, providing a good starting template for the growth of high-quality YBCO layers. *J*_c_ drastically increased up to a factor of 2 for YBCO/DyBCO multilayer films, compared to YBCO monolayer films in both the self-field and applied magnetic field. The significant improvement in *J*_c_ is attributed to the improvement of surface smoothness and enhanced flux pinning properties as revealed by the magnetic-field angular dependence of *J*_c_. Schematic illustration of the YBCO/DyBCO multilayers is presented in Figure 5a.

S Kang et al. [114] prepared multilayer structures comprising YBCO films with self-assembled BZO nanodots with interlayers of CeO_2_ grown on rolling-assisted biaxially textured substrates (RABiTSs) using PLD. Enhancement of pinning in the multilayers was attributed to the presence of columnar defects comprised of self-assembled nanodots of BZO as well as planar CuO-type stacking defects arising as a result of interfacial reactions in the multilayers. The corresponding cross-section TEM micrograph of YBCO + BZO/CeO_2_ multilayer is shown in Figure 5b,c.

T. Haugan et al. [115] applied a different approach in PLD and succeeded in introducing the non-superconducting phase Y_2_BaCuO_5_ (Y211) into YBCO films in a controlled manner. By using two different targets (YBCO and Y211) and alternately depositing a YBCO film layer and a discontinuous Y211 film layer, a pancake-like array of precipitates was formed in the obtained film matrix. In this case, either *H* is parallel to the *c*-axis or the *ab*-plane, vortex pinning efficiency is improved significantly [116]. Moreover, recent studies have also shown that these Y211 nanoparticles are not only effective in enhancing *J*_c_ but also in reducing its anisotropy [117].

### 3.4. Three-Dimensional APCs (3D APCs)

To obtain better performance, the ideal materials need to be carefully selected when introducing the secondary phase to the HTS matrix. In terms of pinning efficiency, the spherical secondary phase needs to maintain the proper size and shape, and is required to be uniformly distributed among the superconducting matrix, which is necessary. Therefore, it is not an easy task to find a secondary phase material that will persist in ideal presence and distribution during superconductor synthesis as APCs. At present, many compounds have been applied to investigate the possibility of becoming effective APCs.

#### 3.4.1. Introduction of 3D APCs by Vapor Deposition Methods

Vapor deposition methods have been used to prepare spontaneously generated and segregated spherical vortex pinning centers, including Y_2_O_3_ [118,119,120,121,122,123], Y211 [124,125], BaIrO_3_, Ag, Au [126], and Gd_2_Ba_4_CuWO_y_ compound. When HTS films are deposited using techniques such as CVD or PVD, the relationship between the orientation of APCs and superconducting matrix is often determined by the growth mechanism of both, leading to a fixed overall orientation of APCs, since the secondary phase and the superconducting matrix nucleated simultaneously [127].

**Figure 6:**
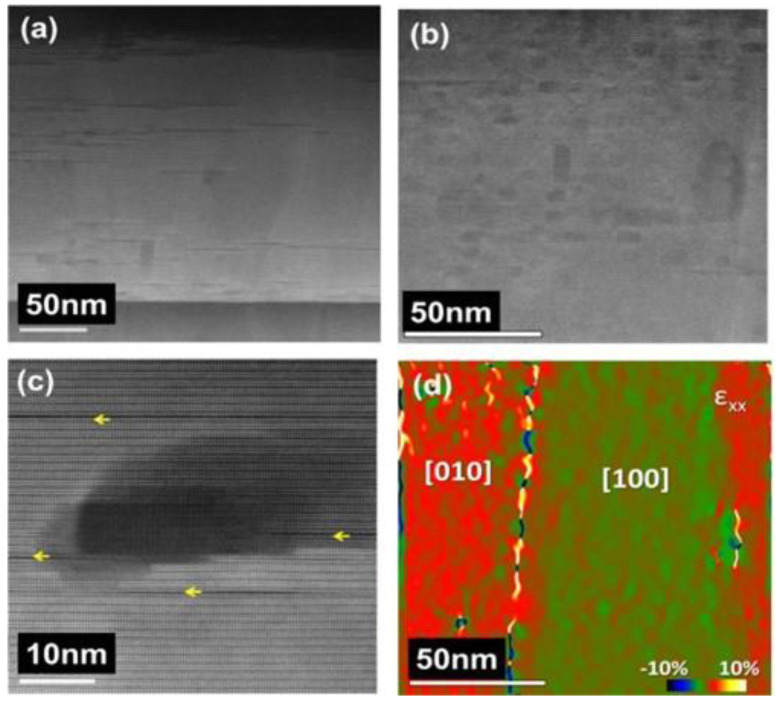
(**a**,**b**) Low- and high-magnification cross-sectional TEM image of the YBCO + 5.44A% Y_2_O_3_ thin film. (**c**) High-resolution TEM image of a Y_2_O_3_ nanoparticle embedded in a superconducting matrix. (**d**) ε_xx_ deformation map of (**b**). Reprinted with permission from Ref. [128]. Copyright 2015, IOP Publishing.

S. K. Viswanathan et al. [123] prepared Y_2_O_3_/YBCO nanocomposite films by PLD, which possessed high *J*_c_ up to five times than that of pure YBCO films in high magnetic field. In addition, their results demonstrated that the size, interparticle spacing, and density of Y_2_O_3_ nanoparticles could be tailored by varying the number of laser pulses in order to determine the optimum size for effective immobilization of vortices. Paolo Mele et al. [128] deposited 3D Y_2_O_3_-added YBCO films on SrTiO_3_-buffered MgO substrates by PLD using surfaced-modified YBCO targets. The 5.44A% Y_2_O_3_-added sample presents a very high value of pinning force (*F*_p,max_~14.3 GN/m^3^), approaching the value obtained in YBCO films with added 1D BaZrO_3_ nanorods, but with less depression in *T*_c_. The corresponding TEM analysis of the nanocomposite film is shown in Figure 6. Masashi Miura et al. [129] applied the LTG-PLD technique to prepare “Sm_1+x_Ba_2-x_CuO_y_+nanoparticle” films. Low *T*_c_ 3D nanoparticles are randomly dispersed in the superconducting matrix due to the local compositional fluctuation, and significant improvement in the pinning properties has been achieved. The SmBCO nanocomposite films obtained an extremely high *J*_c_~0.1 MA/m^2^ (77 K, *B/*/c, *B* = 8 T).

#### 3.4.2. Introduction of 3D APCs by Liquid Phase Deposition Methods

There are two routes for introducing 3D APCs by liquid phase chemical preparation: in the first case, named “*in situ*”, a complex solution containing all elements is prepared to form HTS films and dopants. By this way, APCs are spontaneously generated and segregated during the HTS films growth. The second case, called “ex situ”, involves dispersing preformed oxide nanoparticles (NPs) added to YBCO precursor solution to define and strictly control NPs size, concentration and composition before the HTS film growth occurs. In this way, it is possible to form point-like or spherical vortex pinning centers, which are expected to be very effective at low temperatures and high-field regimes [130].

During the spontaneously generated and segregated chemical preparation of HTS films, some of elements of APCs can react with precursors, superconducting substrates, or other additives. Taking advantage of this feature, organic (trifluoroacetate [93,131], ethoxide [132], acetylacetonate [131], acetates [133,134], naphthenate [135,136]) or inorganic (nitrates [133,137], titanate [138]) salts corresponding to elements of APCs are added during the synthesis stage of HTS films, and APCs are spontaneously generated and widely distributed in HTS films during the sintering process.

The superconducting properties of HTS films with different types of APC and different deposition methods are shown in Table 1.

**Figure 7:**
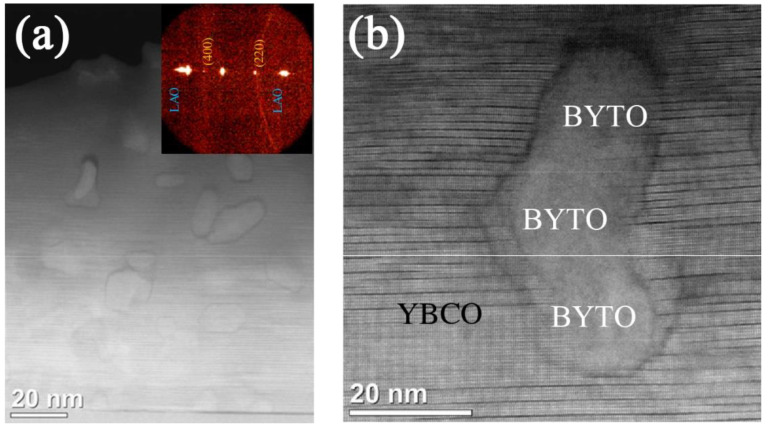
(**a**) Low and (**b**) high magnification Z-contrast image of BYTO nanoparticles embedded in the YBCO matrix. Reprinted with permission from Ref. [151]. Copyright 2014, IOP Publishing.

Compared with other methods, the introduction of APCs by in situ chemical methods has significant advantages such as low complexity and low cost, but there are disadvantages such as the uncertain orientation, non-uniform size, easy agglomeration, and easy chemical reaction with the superconducting matrix. For example, in the preparation of spontaneously segregated Ba_2_YTaO_6_ nanoparticles in YBCO films, there is the coalescence of three nanoparticles, as shown in Figure 7. The aggregated randomly oriented nanoparticles expose less interfacial area to the epitaxial YBCO matrix, i.e., less incoherent interface than three separate nanoparticles [151]. In this scenario, the nanoparticles are much less efficient in terms of generating nanostrained areas in YBCO matrix and cannot significantly improve *J*_c_ performance under external magnetic fields [151]. In addition, it should be emphasized that the advantage of introducing 3D APCs (either by in situ or ex situ route) is an enhanced pinning performance in magnetic fields, especially at temperatures lower than 77 K through the effect of randomly dispersed strongly correlated defects. In this case, the angular dependence of *J*_c_ is isotropic and the strong *c*-axis correlated peak is absent [152].

**Figure 8:**
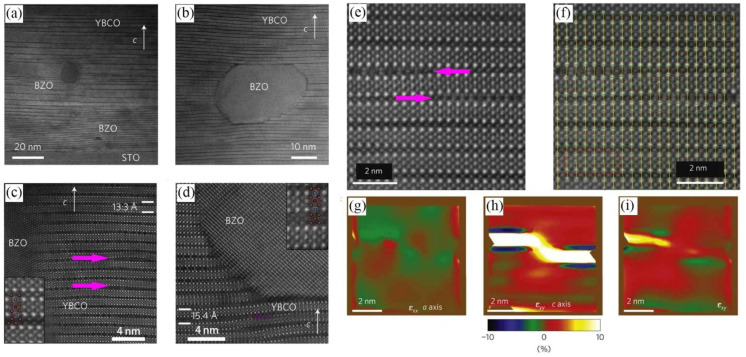
(**a**,**b**) HAADF images of BaZrO_3_ nanoparticles in the YBCO matrix. (**c**,**d**) High-resolution HAADF images of the region between two BaZrO_3_ nanoparticles. (**e**) HAADF image where the strain maps are obtained. (**f**) Grid obtained by PPA (Peak Pairs Analysis) from the image shown in (**e**). (**g**–**i**) ɛ_xx_, ɛ_yy_ and ɛ_xy_ maps, respectively. Reprinted with permission from Ref. [131]. Copyright 2012, Nature.

Recently, V Rouco et al. [153] have shown that solution-derived YBCO nanocomposites can reach extraordinary pinning forces at 77 K, thanks to the isotropic nanostrain introduced by nanoparticles. The presence of random nanoparticles during the YBCO growth induces a high density of stacking faults (double or triple CuO chain layers) in the film as a mechanism to relax the incoherent interfaces. Very efficient artificially induced isotropic core pinning in CSD nanocomposites is attributed to Cooper pair suppression in nanostrained regions surrounding the stacking faults in the form of partial dislocations [33,131]. Llordés A et al. [131] studied the behavior of chemically inert BaZrO_3_ spherical nanoparticles when used as APCs and concluded that the introduction of BaZrO_3_ secondary phase leads to the generation of various defects in the YBCO matrix (as shown by the pink arrows in Figure 8a–d). Numerous stacking faults and Y248 phases nucleated at the junction of YBCO and BaZrO_3_, and the nanoscale strain resulting from these defect generation (as shown in Figure 8e–i) suppressed the generation of Cooper pairs, thus improving the vortex pinning and ultimately the superconductivity.

In addition to the common binary or ternary oxides, inert oxides from the same *RE*BCO system (*RE* = Sm, Gd, Eu, etc.) can also be used as vortex pinning centers. For example, in YBCO system, the Y-2411-*M* phase (Y_2_Ba_4_Cu*M*O_x_, *M* = Nb, Ta, Mo, W, Ru, Zr, Bi, and Ag) is also a promising material. Similarly, inert phases with chalcogenide structures that are stable at high temperatures, such as Ba_2_*RE*TaO_6_, Ba_2_Y(Nb, Ta)O_6_, also have been reported as APCs [84,154].

#### 3.4.3. Substrate Decoration by 3D APCs

Modifying substrate surface by 3D APCs before depositing superconducting films was one of the first methods to introduce APCs into HTS films. The modification of substrate surface is accomplished by growing different types of nanoparticles such as metals [155,156,157,158] or oxides [138,159,160,161,162,163,164] on substrates to create interfacial defects. Due to the presence of these nanoparticles at the phase interface, the deposited YBCO crystalline surface is distorted or flexed above the nanoparticles, producing low-angle grain boundaries or dislocations that may extend over the entire film thickness.

**Figure 9:**
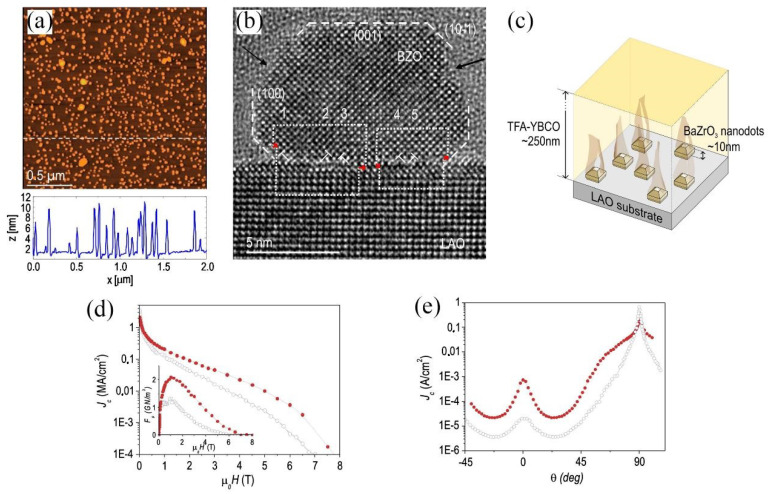
(**a**,**b**) AFM and TEM images of BaZrO_3_ nanoparticles. (**c**) Schematic drawing of the 3D configuration of the YBCO film grown on a nanostructured BaZrO_3_/LaAlO_3_ template. (**d**) *J*_c_ vs. *H* applied along the *c*-axis (*θ* = 0°) and 77 K for a standard film (open symbols) and a nanostructured one (closed symbols). (Inset) the respective pinning forces determined from *J*_c_(*H*). (**e**) Angular dependence of *J*_c_ at 77 K and 7 T for a standard film and a nanostructured film. Reproduced with permission from [169]. Copyright 2011, Elsevier.

PLD and MOD (Metal–Organic Decomposition) technique has been used for the modification of substrates before growing YBCO films [165,166,167,168]. Patricia Abellan et al. [169] elucidated the effect of a BaZrO_3_ nanoparticle template on the microstructure and *J*_c_ of YBCO films. Figure 9a,b shows the AFM and TEM images of the nanostructured interface of BaZrO_3_/LaAlO_3_; Figure 9c presents a schematic drawing of the 3D configuration of a YBCO film grown on a nanostructured BaZrO_3_/LaAlO_3_ template; Figure 9d,e displays the corresponding *J*_c_ measurement results.

Matsumoto et al. [170] reported the introduction of Y_2_O_3_ nanoparticles at the phase interface between the substrate and film. Physical property tests showed that when *H* is parallel to *c*-axis there was a significant enhancement in *J*_c_ properties, indicating that the introduction of Y_2_O_3_ nanoparticles resulted in enhanced vortex pinning. In another report by Aytug et al. [156], the enhancement of *J*_c_ was observed by treating SrTiO_3_ substrates with Ir nanoparticles. The authors noted that YBCO planes growing on top of Ir nanoparticles were curved and produced random pinning. In general, random pinning is generated due to the uniform distribution of point defects within the entire film volume. In this case, the film thickness is 100 ~ 200 nm and the strain field running through the entire film thickness surrounds the entire periphery of the Ir nanoparticles. The difference in the pinning mechanism between Y_2_O_3_ and Ir nanoparticles can be explained by their reaction with the YBCO film through the chemical reaction with the YBCO phase. When Ir is present at the SrTiO_3_/YBCO interface, these Ir nanoparticles may have partially reacted chemically with YBCO, and the resulting volume change may provide an alternative way to relieve strain, thus reducing the driving force for dislocation formation and weakening the associated magnetic flux pinning ability. However, the Y_2_O_3_ nanoparticles are chemically inert with YBCO, and thus they are intact at the substrate and film phase interface. A. Crisan et al. [155,171] deposited Ag nanodots on SrTiO_3_ substrates by PLD prior to the growth of (Cu, Tl)BaSrCa_2_Cu_3_O_y_ films. The results show that the Ag nanodots increased the *J*_c_ more than one order of magnitude than that without nanodots. M. Ionescu et al. [172] also applied Ag nanodots to decorate the YSZ substrate. Under magnetic fields, *J*_c_ was three times higher for the YBCO film grown on the modified YSZ surface than the virgin surface.

### 3.5. Hybrid 1D + 3D APCs

The 1D columnar APCs perform very well in enhancing *J*_c_, but one of the shortcomings is that the performance of *J*_c_ degrades more with the change of direction of applied external magnetic fields. In addition, at higher temperatures, due to thermal excitation, the vortex tends to form a double kink structure, and even if they contain crystal defects in the *c*-axis direction, the unpinned vortices can still move due to the Lorentz force, resulting in degraded performance. To solve this problem, combinations of APCs with different dimensions have been developed. It has been shown that the simultaneous formation of 1D and 3D APCs can effectively compensate for the lack of performance of 1D columnar APCs only, adapting to applied magnetic fields with different applied directions [173].

Mele et al. [152] reported the combined application of two different types of pinning centers to successfully introduce both BaZrO_3_ columns and Y_2_O_3_ nanoparticles into YBCO films using the PLD technique. Although *J*_c_ increased only slightly in the intermediate angular region, the significant decrease of *J*_c_ with angle in *c*-axis direction was significantly improved compared to YBCO films with only BaZrO_3_ nanocolumns added. Similar results were obtained in a related study by Ding F. Z et al. [174]. Subsequently, combinations of columns with nanoparticles of different materials were also reported to enhance the *J*_c_ performance of YBCO films, sufficiently reducing the anisotropy of *J*_c_ [65,77,149,175]. For example, BaSnO_3_ columns and Y_2_O_3_ nanoparticles were tried in combination, which significantly enhanced *J*_c_ and reduced the anisotropy of *J*_c_ [64,175]. TEM studies of YBCO + 3%BaSnO_3_ and YBCO + 3%BaSnO_3_ + Y_2_O_3_ nanocomposite films showed that only columnar nanostructures were formed in YBCO + BaSnO_3_ films, while YBCO + BaSnO_3_ + Y_2_O_3_ thin films formed both columnar and spherical nanostructures.

**Figure 10:**
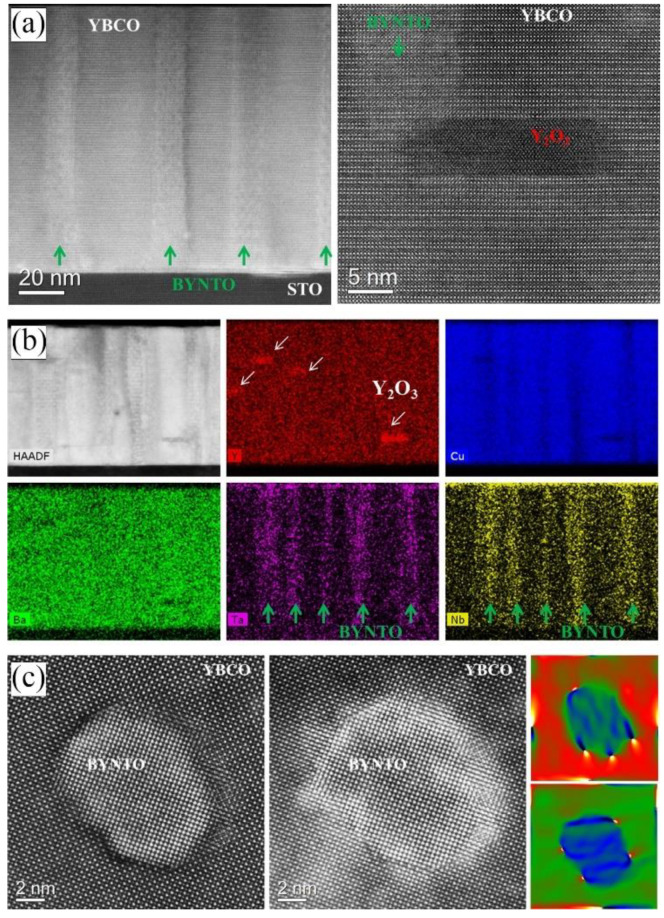
TEM images of YBCO-BYNTO films deposited at a growth rate of 0.02 nm s^−1^. (**a**) Cross-section view. Left panel: HAADF overview showing the BYNTO straight columns; right panel: high-resolution HAADF image of BYNTO nanocolumn decorated by Y_2_O_3_ nanoparticle. (**b**) Cross section view: HAADF image of the mapped area together with elemental Y, Ba, Cu, Ta, and Nb EDX maps. (**c**) Plan view. Left and central panels: HAADF and LAADF images. Interphase dislocations causing local strain can be visualized by GPA maps (right panel). Reprinted with permission from Ref. [148]. Copyright 2020, IOP Publishing.

In 2016, F. Rizzo et al. [146] deposited YBCO films with pinning additions of 5 at. % Ba_2_YTaO_6_ (BYTO) by PLD technique. Excellent vortex pinning at 77 K was obtained with remarkably high irreversibility fields greater than 10 T, representing the highest ever achieved values in YBCO films. Lately, in 2020, G Celentano et al. [148] deposited YBCO films with pinning additions of 2.5% Ba_2_YTaO_6_ + 2.5% Ba_2_YNbO_6_ (BYNTO) double-perovskite secondary phases by PLD technique in an extended film growth rate, *R* = 0.02–1.8 nm s^−1^. This microstructure results in very efficient vortex pinning at 77 K, leading to a remarkable improvement in *J*_c_ behavior, with *F*_p max_ ~ 13.5 GN m^−3^ and *H*irr ~ 11 T. The corresponding TEM images are shown in Figure 10.

Other combinations of different types of columns and nanoparticles have also been introduced into YBCO films, such as BaSnO_3_ columns and Y211 nanoparticles [149], BaZrO_3_ columns and BaCeO_3_ nanoparticles [150], BaHfO_3_ columns and Y_2_O_3_ nanoparticles [176], all of which induced excellent *J*_c_ performance.

### 3.6. Ferromagnetic APCs

In the study of vortex pinning in HTS films, the choice of APCs has generally been made for insulating nonmagnetic metal or oxide. However, there have been successful attempts to introduce ferromagnetic APCs into YBCO films. Therefore, we listed ferromagnetic APC as a separate section to discuss.

**Figure 11:**
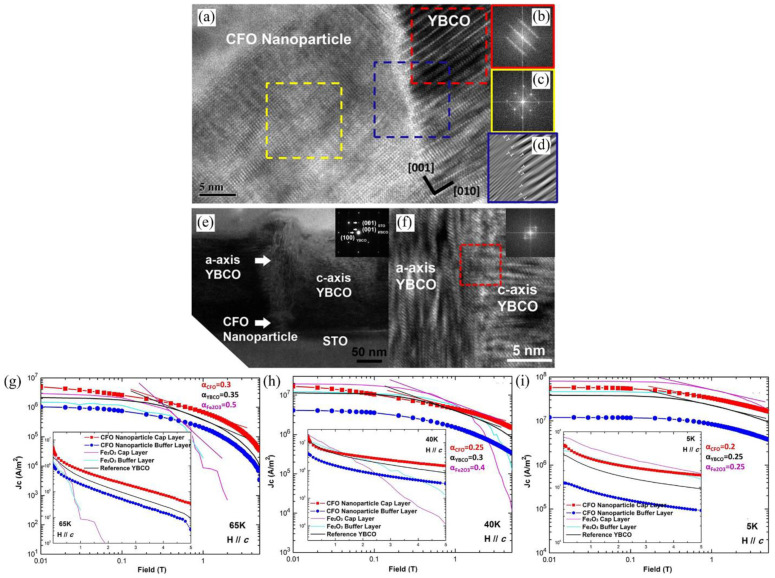
(**a**) HRTEM image of the CoFe_2_O_4_/YBCO heterointerface. (**b**,**c**) FFT diffraction of YBCO and CoFe_2_O_4_ phases. (**d**) IFFT of YBCO/CoFe_2_O_4_ interface. (**e**) TEM image with the corresponding SAED. (**f**) HRTEM image of YBCO(001)/YBCO(010) interface with corresponding FFT diffraction. *J*_c_ vs. *H* measured at (**g**) 65 K, (**h**) 40 K, and (**i**) 5 K. Reprinted with permission from Ref. [180]. Copyright 2013, IEEE-Inst Electrical Electronics Engineers Inc.

Before depositing YBCO films, J. Wang and C.F. Tsai et al. prepared the Fe_2_O_3_ film on HTS films as a cover layer [177,178]. The measurements showed a significant enhancement of *J*_c_ for samples consisting of the Fe_2_O_3_ overlayer. Moreover, ferromagnetic nano-inclusions such as YFe_3_O_4_ [179] (YFO) and CoFe_2_O_4_ [180] (CFO) were also successfully applied to enhance the vortex pinning properties of YBCO films. By introducing ferromagnetic CoFe_2_O_4_ nanoinclusions, both magnetic and defect pinning can be incorporated into YBCO films to achieve stronger pinning effects under higher applied magnetic field regimes, as shown in Figure 11. The results suggest that the CFO nanoparticle cap is an ideal approach to introduce ordered vortex pinning centers in YBCO films, which shows enhanced superconducting properties (as shown in Figure 11g–i) both at self-field and under high applied field regimes without degrading YBCO’s intrinsic properties.

At sufficiently low concentrations, Fe incorporation into the YBCO matrix can effectively form vortex pinning; with increasing Fe concentration, the so-called “poisoning effect” can be observed [181]. The enhanced vortex pinning properties of YBCO films containing ferromagnetic nano-inclusions were discussed using a Lorentz force reduction mechanism, which is effective for the low applied magnetic field region (~1 T) [182]. Ferromagnetic La_0.7_Sr_0.3_MnO_3_ (LSMO) was deposited on top of SrTiO_3_ substrates using a PLD approach, followed by the deposition of YBCO films. The physical test results showed that modified samples exhibit stronger *J*_c_ performance than unmodified samples, which is due to the presence of LSMO nanoparticles at the YBCO/SrTiO_3_ interface that creates structural defects such as penetration dislocations, which lead to vortex pinning in the *c*-axis direction in YBCO films. Additionally, the *J*_c_ enhancement of YBCO films with modified substrates can also be understood by the Lorentz force reduction pinning mechanism [182]. Jijie Huang et al. [183] incorporated vertically aligned nanocomposite (VAN) (La_0.7_Sr_0.3_MnO_3_)_0.5_(CeO_2_)_0.5_ and pure La_0.7_Sr_0.3_MnO_3_ layers into YBCO films as bilayer stacks for vortex pinning enhancement. The films showed high epitaxial quality, demonstrated by XRD and TEM studies. The *T*_c_ of the bilayers is about 90 K, which is close to that of pure YBCO films, while both the self-field *J*_c_^sf^ and in-field *J*_c_^in-field^ are largely enhanced. Among all samples, the film with VAN caplayer showed the highest *J*_c_ values in all field ranges. This study demonstrated an effective way towards the tunable pinning effect for YBCO coated conductors by both defect and magnetic pinning.

## 4. Summary and Outlook

The large-scale application of HTSs in energy, transportation, information, space exploration and other fields will bring huge economic benefits. At present, the application of superconductivity is not only in the conceptual stage, but also has been fully proved to be feasible. The emergence of new driving forces, such as the performance, reliability, practicability, cost performance, and competitiveness of existing technologies, is the research focus of future large-scale superconducting practical application technology.

This paper focuses on the types of APCs in HTS films, the methods of introducing, and the principles of performance enhancement. Although the general idea of vortex pinning is simple, the theory behind the successful exploitation of magnetic flux pinning center behavior still proves to be very challenging. Depending on the deposition techniques and the intended applications of the product, different dimensions, morphologies, orientations and concentrations of APCs need to be studied. Combining the current state of research, it seems that for HTS films, the best overall approach is a combination of 1D (columnar secondary phase and defects) and 3D (nanoparticles). This combination provides high performance while allowing a slightly larger margin of error, considering that the orientation of the 1D pinning center is only a contribution to the total pinning in the *RE*BCO layer. Moreover, the difficulty of the current research is that the vortex pinning theory of superconductivity lags behind the experimental progress and cannot completely explain and guide the next research on superconductivity preparation and application work at the theoretical level.

## Figures and Tables

**Table 1:** The types, dimension, preparation methods of APCs and corresponding thickness, *J*_c_, *T*_c_, and *F*_p_^max^ of YBCO nanocomposite films.

APC	Film	Method	Dimension	Film Thickness (nm)	*J*_c_ (MA/cm^2^)	*T*_c_ (K)	*F*_p_^max^ (GN m^−3^)	References
HfO_2_	YBCO	*ex situ* Ink-Jet Printing	3D	450–500	3.1 (77 K, 0 T)	—	6.8 (77 K, 0 T)	[139]
Nanodiamond	YBCO	*ex situ* MOD	3D	75	4 (77 K, 0 T)	90.4 ± 0.2	—	[130]
BaZrO_3_	YBCO	*ex situ* MOD	3D	260	4.7 (77 K, 0 T)	91.3	4.1 (77 K, 1 T)	[140]
SrZrO_3_	YBCO	*ex situ* MOD	3D	290	2.7 (77 K, 0 T)	90.2	2.0 (77 K, 1 T)	[140]
BaHfO_3_	YBCO	*ex situ* MOD	3D	240	4.5 (77 K, 0 T)	90.7	5.9 (77 K, 1 T)	[140]
BaTiO_3_	YBCO	*ex situ* MOD	3D	300	4.6 (77 K, 0 T)	92.3	1.5 (77 K, 1 T)	[140]
ZrO_2_	YBCO	*ex situ* CSD	3D	180	5 (77 K, 0 T)	89.6	6 (77 K, 0 T)	[141]
MnFe_2_O_4_	YBCO	*ex situ* CSD	3D	150 ± 10	2 (5 K, 0 T)	76	—	[142]
BaZrO_3_	YBCO	*in situ* MOD	3D	200–270	2.2 (77 K, 1 T)	91	21 (77 K, 2 T)	[93]
Ba_2_YTaO_6_	YBCO	*in situ* CSD	3D	250	4–5 (77 K, 0 T)	89–90	6 (77 K, 1 T)	[132]
Y_2_O_3_	YBCO	*in situ* PLD	3D	—	2.62 (44 K, 0 T)	89.26	7.8 (77 K, 2 T)	[118]
Ba_2_LuNbO_6_	YBCO	*in situ* PLD	1D	160	0.76 (77 K, 2 T)	89.5	17.5 (77 K, 1.5 T)	[143]
BaHfO_3_	YBCO	*in situ* PLD	1D	190	0.61 (77 K, 0 T)	89.0	15.9 (77 K, 3 T)	[144]
BaSnO_3_	YBCO	*in situ* PLD	1D	220	0.64 (77 K, 1 T)	89.6	13.4 (77 K, 2 T)	[144]
BaSnO_3_	YBCO	*in situ* PLD	1D	271–310	0.38 (77 K, 5 T)	88.6	28.3 (77 K, 2 T)	[71]
Ba_2_YNbO_6_	YBCO	*in situ* PLD	1D	500–1000	4.5 (75.6 K, 0 T)	92.0–92.5	32.3 (75.5 K, 0 T)	[77]
Ba_2_Y(Nb/Ta)O_6_	YBCO	*in situ* PLD	1D	250	1.18 (77 K, 2 T)	90.5–90.8	21.5 (77 K, 1.7 T)	[78]
Ba_2_YNbO_6_	YBCO	*in situ* PLD	1D	400	1.6 (77K, 0 T)	88–91	6 (77 K, 1.4 T)	[145]
Ba_2_YTaO_6_	YBCO	*in situ* PLD	1D	215–230	1.6 (77K, 0 T)	—	9.1 (77 K, 3 T)	[146]
Ba_2_Y(Nb/Ta)O_6_	YBCO	*in situ* PLD	1D	215–230	3.2 (77 K, 1 T)	—	11.5 (77 K, 4.4 T)	[146]
Ba_2_YNbO_6_	YBCO	*in situ* PLD	1D	200–800	4.1 (77 K, 0 T)	87.4–88.3	—	[79]
BaZrO_3_	(YCa)BCO	*in situ* PLD	1D	160–170	4.39 (77 K, 1 T)	87.5	98 (65 K, 0 T)	[147]
Ba_2_YTaO_6_ + Ba_2_YNbO_6_	YBCO	*In situ* PLD	1D + 3D	150–230	7.5 (10 K, 0 T)	89.9	900 (10 K, 12 T)	[148]
BaSnO_3_ + Y_2_BaCuO_5_	YBCO	*In situ* PLD	1D + 3D	—	3.4 (77 K, 0 T)	—	9.65 (77 K, 0 T) 44.7 (77 K, 0 T)	[149]
BaZrO_3_ + BaCeO_3_	YBCO	*In situ* PLD	1D + 3D	310–380	4.5 (77 K, 8 T)	87.7–88.9	—	[150]
